# Analysis of current status and influencing factors of fear of recurrence in patients with coronary heart disease after PCI: A cross-sectional study

**DOI:** 10.1097/MD.0000000000047655

**Published:** 2026-03-06

**Authors:** Qin Peng, Chaofeng Li, Yuxuan Shao, Xinyi Hou, Mengdie Hu

**Affiliations:** a School of Nursing, Nanjing University of Chinese Medicine, Nanjing, Jiangsu, China; b The Second Affiliated Hospital of Nanjing University of Chinese Medicine, Nanjing, Jiangsu, China.

**Keywords:** coronary heart disease, fear of recurrence, influencing factors, nursing, post-PCI

## Abstract

This study aims to investigate the current status and influencing factors of fear of recurrence (FoR) in patients with coronary heart disease (CHD) after percutaneous coronary intervention, providing reference for developing targeted intervention measures. A convenience sampling method was used to select 139 CHD patients who underwent percutaneous coronary intervention. A general information questionnaire, the Chinese version of Fear of Progression Questionnaire–Short Form, Brief Illness Perception Questionnaire, Perceived Social Support Scale, and 10-item Center for Epidemiological Studies Depression Scale were used for investigation. The median Fear of Progression Questionnaire–Short Form score was 31 (28, 34), with an FoR incidence of 33.1%. Logistic regression analysis showed that unemployment, first surgery, severe depression, and low perceived social support were risk factors for FoR (*P* < .05). CHD patients generally experience varying degrees of FoR. Special attention should be paid to those with depressive tendencies, unemployment, first surgery, and low perceived social support. Targeted assessment and intervention are needed to help patients overcome FoR and promote rehabilitation.

## 1. Introduction

According to the China Cardiovascular Health and Disease Report 2023,^[1]^ the number of coronary heart disease (CHD) patients in China has reached 11.39 million, seriously affecting quality of life and imposing a heavy burden on healthcare systems.^[2]^

Percutaneous coronary intervention (PCI), as a minimally invasive revascularization technique, has revolutionized the management of CHD over the past 3 decades.^[3]^ By deploying stents to dilate stenotic coronary segments, PCI rapidly restores myocardial blood flow, alleviates ischemic symptoms, and reduces short-term mortality in patients with acute coronary syndromes or refractory stable angina.^[4]^

PCI has become the primary revascularization method for CHD.^[5]^ However, PCI cannot provide permanent improvement, and there remains a risk of vascular restenosis.^[6,7]^ Within 1 year after surgery, 20% to 30% of patients experience adverse cardiac events, such as restenosis or myocardial infarction,^[8]^ leading to high readmission rates. Beyond the physical consequences of recurrent disease, post-PCI patients often face significant psychological distress, with fear of recurrence (FoR) emerging as a prominent and underrecognized issue.^[9]^ Consequently, post-PCI patients are prone to FoR and progression. FoR refers to the fear of adverse physiological, psychological, and social consequences arising from disease progression or the recurrence of the disease itself.^[10]^

Relevant studies^[11]^ have found high levels of FoR among elderly outpatients with CHD, highlighting the importance of addressing this issue. Therefore, this study investigates the current status and influencing factors of post-PCI FoR to provide reference for personalized clinical management.

## 2. Materials and methods

### 2.1. Participants and procedure

A cross-sectional study was conducted in a tertiary hospital in China between October 2024 and May 2025. A convenience sample of 139 patients who underwent PCI for CHD and were hospitalized in the Cardiology Department was enrolled in this study.

#### 2.1.1. Participants

Inclusion criteria were as follows: meeting CHD diagnostic criteria, stable condition 48 to 72 hours after PCI, clear consciousness and communication ability, and signed informed consent.

Exclusion criteria included inability to complete questionnaires, severe comorbidities or malignancies, and history of mental illness.

This study was approved by the Medical Ethics Committee of the Second Hospital of Nanjing (2025-LS-ky-012). The research team approached the eligible patients in the ward and explained the study’s purpose, procedure, benefits, and risks. After obtaining informed consent from each participant, the research team distributed the paper-based questionnaires to the patients, who completed the questionnaires independently. This study has 12 independent variables. The sample size should be at least 5 to 10 times the total number of independent variables, while also accounting for a 10% nonresponse rate. Calculations indicated a sample size of 70 to 134 cases. One hundred forty-five questionnaires were distributed throughout the study, and 139 valid questionnaires were collected, resulting in an effective response rate of 95.87%.

### 2.2. Measures

#### 2.2.1. General information questionnaire

Developed through literature review and expert discussion, including 9 items on demographics (gender, age, education, residence), social factors (occupation, medical payment), and disease-related factors (comorbidities, first surgery status, triglyceride levels).

#### 2.2.2. Fear of Progression Questionnaire–Short Form

Developed by Mehnert et al^[12]^ and Chinese version validated by Wu et al,^[13]^ this 12-item scale uses a 5-point Likert scale (1 = “never” to 5 = “always”), with total scores ranging from 12 to 60. Scores ≥34 indicate abnormal FoR,^[14]^ and Cronbach α = 0.883.

#### 2.2.3. Brief Illness Perception Questionnaire (BIPQ)

Developed by Broadbent et al^[15]^ and validated in Chinese by Mei et al^[16]^, this 9-item scale assesses illness perception. Items 1 to 8 use 0 to 10 scoring (reverse scoring for items 3, 4, and 7), with higher scores indicating greater perceived disease threat. Cronbach α = 0.77.

#### 2.2.4. Perceived Social Support Scale

Compiled by Blumenthal et al,^[17]^ this 12-item scale with 3 dimensions (family, friend, and other support) uses a 7-point Likert scale. Total scores range from 12 to 84, with higher scores indicating better social support. Cronbach α = 0.83.

#### 2.2.5. 10-item Center for Epidemiological Studies Depression Scale

Developed by Andresen et al,^[18]^ the Chinese version^[19]^ has good reliability and validity for measuring depression in middle-aged and elderly populations. Each item is scored on a scale of 0 to 3 points, with two items scored in reverse, total scores range from 0 to 30, with scores ≥10 indicating depressive symptoms.^[20]^

### 2.3. Statistics

Data analysis was performed using SPSS 27.0 software (International Business Machines Corporation [IBM Corp], Armonk). Normally distributed quantitative data were described using mean ± standard deviation, and comparisons between 2 groups were conducted using the independent samples *t* test. Non-normally distributed quantitative data were described using median and interquartile range and analyzed using the nonparametric Mann–Whitney *U* test. Categorical data were described using frequency and proportion. Univariate analysis was performed using the chi-square test, while multivariate analysis employed binary logistic regression. A *P* value < .05 was considered statistically significant.

## 3. Results

### 3.1. General characteristics

Table 1 shows the sociodemographic and clinical characteristics of the participants. Among the 139 patients, 105 were male (75.5%) and 34 female (24.5%). Moreover, the majority of participants were aged 60 or older, with 84 individuals falling into this category (60.4%). Other characteristics included occupational status: be on the job, 27 cases (19.4%); retire, 69 cases (49.6%); individuals or farmers, 14 cases (10.1%); unemployed, 29 cases (20.9%); education: primary school or below, 44 cases (31.7%); junior high school, 50 (36.0%); senior high/technical secondary school, 28 (20.1%); college or above, 17 (12.2%); residence: rural, 60 (43.2%); town, 79 (56.8%); medical insurance: employee, 67 (48.2%), resident, 67 (48.2%), self-paying, 5 (3.6%); comorbidities: <4 types, 90 (64.7%), ≥4 types, 49 (35.3%); first surgery: yes, 56 (40.3%); no, 83 (59.7%); and triglyceride levels: <1.4 mmol/L, 70 (50.4%); ≥1.4 mmol/L, 69 (49.6%).

**Table 1:** Demographic characteristics and clinical characteristics of patients after PCI (n = 139).

Variable	N (%)
Gender	
Male	105 (75.5)
Female	34 (24.5)
Age (yr)	
<45	10 (7.2)
45–60	45 (32.4)
>60	84 (60.4)
Occupational status	
Be on the job	27 (19.4)
Retire	69 (49.6)
Individuals or farmers	14 (10.1)
Unemployed	29 (20.9)
Education level	
Primary school or below	44 (31.7)
Junior high school	50 (36.0)
Senior high/technical secondary school	28 (20.1)
College or above	17 (12.2)
Residence	
Rural	60 (43.2)
Town	79 (56.8)
Number of comorbidities	
<4	90 (64.7)
≥4	49 (35.3)
Medical insurance	
Employee insurance	67 (48.2)
Resident insurance	67 (48.2)
Self-paying	5 (3.6)
Whether the surgery is the first time	
Yes	56 (40.3)
No	83 (59.7)
Triglyceride levels (mmol/L)	
<1.4	70 (50.4)
≥1.4	69 (49.6)

PCI = percutaneous coronary intervention.

### 3.2. Descriptive analysis of study variables

Table 2 shows the range and mean scores of variables. The Fear of Progression Questionnaire–Short Form score was 31 (28, 34), the perceived social support score was 59.54 ± 6.077, the BIPQ score was 40 (37, 44), and the 10-item Center for Epidemiological Studies Depression Scale score was 9 (8, 12).

**Table 2:** Description of FoP, social support, illness perception, and depression scores (n = 139).

Variables	Range	Mean
FoP	12–60	31 (28, 34)
Perceived social support	12–84	59.54 ± 6.077
Brief illness perception	0–80	40 (37, 44)
Depression	0–30	9 (8, 12)

FoP = fear of progression.

### 3.3. Univariate analysis of factors influencing FoR in patients with CHD after PCI

Table 3 shows that among sociodemographic factors, gender, age, and place of residence; among sociological data, employment status and type of medical insurance; and among disease-related data, history of first-time surgery and triglyceride level, all exhibited statistically significant differences in terms of FoR in postoperative patients with CHD (*P* < .05). Additionally, scores of BIPQ, Perceived Social Support Scale, and depression showed statistically significant differences in relation to the occurrence of FoR (*P* < .01).

**Table 3:** Univariate analysis of factors influencing fear of recurrence in patients with CHD after PCI (n = 139).

Item	FoP < 34 (n = 93)	FoP ≥ 34 (n = 46)	Statistic value	*P*
Gender			8.008*	.005
Male	77	28		
Female	16	18		
Age (yr)			19.080*	<.001
<45	4	6		
45–60	21	24		
>60	68	16		
Occupational status			37.946*	<.001
Be on the job	8	19		
Retire	62	7		
Individuals or farmers	9	5		
Unemployed	14	15		
Education level			0.594*	.898
Primary school or below	28	16		
Junior high school	33	17		
Senior high/technical secondary school	20	8		
College and above	12	5		
Residence			13.628*	<.001
Rural	30	30		
Town	63	16		
Medical insurance			7.482‡	.019
Employee insurance	52	15		
Resident insurance	39	28		
Self-paying	2	3		
Number of comorbidities			3.528*	.071
<4	69	21		
≥4	24	25		
Whether the surgery is the first time			21.213*	.002
No	64	19		
Yes	29	27		
Triglyceride levels (mmol/L)				
<1.4	53	17	4.941*	.026
≥1.4	40	29		
BIPQ (scores)	38 (35, 41.5)	44 (40, 53.25)	−5.928†	<.001
CES-D-10 (scores)	8 (7.50, 9.00)	13 (11.00, 15.00)	−8.037†	<.001
PSSS (scores)	60.56 ± 5.61	56.57 ± 6.15	1.024§	<.001

BIPQ = Brief Illness Perception Questionnaire, CES-D-10 = 10-item Center for Epidemiological Studies Depression Scale, CHD = coronary heart disease, FoP = fear of progression, PCI = percutaneous coronary intervention, PSSS = Perceived Social Support Scale.

* χ^2^ value.

† *Z* value.

‡ χ^2^ Fisher explicit value.

§ *t* value.

### 3.4. Multivariate logistic regression analysis of factors influencing FoR in patients with CHD after PCI

Whether the occurrence of FoR serves as the dependent variable, statistically significant variables from the univariate analysis are included as independent variables in the binary logistic regression model. Continuous variables were entered as original values; the variable assignments are shown in Table 4.

**Table 4:** Assignment methods of independent variables.

Independent variable	Assignment method
FoP	No = 0, yes = 1
Age (yr)	<45 = 0, 45 to 60 = 1, >60 = 2
Gender	Male = 0, female = 1
Residence	Rural = 0, town = 1
Occupational status	Unemployed = 1, be on the job = 2, retire = 3, individuals or farmers = 4
Medical insurance	Employee insurance = 0, resident insurance = 1, self-paying = 2
Triglyceride levels (mmol/L)	≥1.4 = 0, <1.4 = 1
Whether the surgery is the first time	No = 0, yes = 1
BIPQ (scores)	Enter at original value
PSSS (scores)	Enter at original value
CES-D-10 (scores)	Enter at original value

BIPQ = Brief Illness Perception Questionnaire, CES-D-10 = 10-item Center for Epidemiological Studies Depression Scale, FoP = fear of progression, PSSS = Perceived Social Support Scale.

The results indicated that gender, employment status, place of residence, depression, and perceived social support score were influencing factors for the occurrence of FoR in postoperative patients with CHD, as presented in Table 5.

**Table 5:** Logistic regression analysis of fear of recurrence in patients with CHD after PCI (n = 139).

Independent variable	*Β*	SE	Wald χ^2^	OR	95% CI	*P*
Constant	46.454	19.057	5.942	–	–	.015
Whether the surgery is the first time	−5.013	2.303	4.739	0.007	0.000–0.607	.029
Occupational status (unemployed)	–	–	5.443	–	–	.142
Occupational status (be on the job)	0.209	1.629	0.016	1.232	0.051–30.004	.898
Occupational status (retire)	−5.083	2.243	5.134	0.006	0.000–0.504	.023
Occupational status (individuals or farmers)	−6.406	3.241	3.906	0.002	0.000–0.948	.048
PSSS (scores)	−0.880	0.301	8.545	0.415	0.230–0.748	.003
CES-D-10 (scores)	0.779	0.336	5.359	2.179	1.127–4.213	.021

CES-D-10 = 10-item Center for Epidemiological Studies Depression Scale, CHD = coronary heart disease, CI = confidence interval, OR = odds ratio, PCI = percutaneous coronary intervention, PSSS = Perceived Social Support Scale, SE = standard error.

## 4. Discussion

### 4.1. Prevalence of FoR in patients with CHD after PCI

Research on the FoR among cardiovascular disease patients has not received particular attention thus far, with most studies focusing on patients with various types of cancer,^[21,22]^ for example, studies on breast cancer patients.^[23]^ However, one systematic review indicates^[24]^ that CHD patients will face this psychological issue throughout their long-term disease course. This study found the FoR incidence of 33.1% among post-PCI patients, lower than the 59.5% reported in elderly outpatients^[11]^ but consistent with the findings (35.1%) of Liu et al.^[25]^ The discrepancy may be attributed to differences in age, sample selection, and recovery status – hospitalized patients in the recovery phase may experience reduced FoR as physical function improves.^[24,26]^ The recovery process for CHD is lengthy. Fear of disease progression or anxiety about recurrence can cause significant psychological distress for patients and negatively impact the effectiveness of their rehabilitation. Therefore, healthcare providers should promptly assess the level of fear patients have regarding disease recurrence and understand their psychological state to improve their quality of life and prognosis.

### 4.2. Influencing factors of FoR in patients with CHD after PCI

#### 4.2.1. Occupation status

The results of this study indicate that occupational statuses such as retirees, self-employed individuals, and farmers serve as protective factors against disease recurrence compared with unemployed patients, with *B* values of −5.083 and −6.0406, respectively. This finding aligns with studies on cancer survivors’ research.^[27,28]^ Patients with CHD require long-term anticoagulant therapy following PCI procedures and may also face secondary interventional procedures or other surgical treatments. These treatments necessitate significant financial resources. Given that patients are unemployed and lack stable income sources, research indicates^[29]^ that economic hardship directly amplifies their FoR. Therefore, in clinical nursing practice, patients without income sources can be assessed based on their financial capacity. By collaborating with local charitable organizations, appropriate assistance can be provided to alleviate their economic burden, enhance their confidence in treatment and recovery, and reduce their FoR.

#### 4.2.2. Whether the surgery is the first time

The results of this study indicate that, with patients undergoing their first surgery as the reference group, non-first-time surgery status serves as a protective factor against FoR (*B* = −5.013, *P* < .05). Domestic and international studies^[30,31]^ have found no statistically significant relationship between cancer patients’ fear of disease progression and whether they undergo surgical treatment. This may be because tumor surgery typically involves general anesthesia, whereas PCI procedures often use local anesthesia. During PCI, patients remain conscious and can distinctly perceive pain and fear.^[32]^ Moreover, most patients undergoing their first intervention procedure do so following an acute episode of disease. They often lack systematic understanding of CHD etiology, triggers, and pharmacological management. Insufficient knowledge about postoperative cardiac rehabilitation can lead to psychological amplification of postprocedure discomfort, further intensifying fears of disease recurrence. Therefore, during clinical care, when the patient’s condition permits, preoperative education should be conducted using one-on-one explanation approach. This involves explaining the principles of PCI surgery and potential normal postoperative reactions (such as puncture site pain or transient chest tightness) in plain language. Nonverbal communication methods such as touch and reassurance should be employed to alleviate the patient’s fear and reduce anxiety about disease recurrence.

#### 4.2.3. Depression

The results of this study indicate that depression scores are a risk factor for patients’ FoR. This suggests that the higher a patient’s depression score, the more likely they are to worry about disease recurrence. Higher depression scores correlated with increased FoR, consistent with cancer research.^[33,34]^ Quan et al^[35]^ found that cancer patients’ fear of disease progression was significantly positively correlated with depression; this may be because depressive states foster negative cognitive patterns. Ma et al^[36]^ discovered a significant positive correlation between depression levels and fear of cancer recurrence, with patients scoring higher on the Depression Self-Rating Scale also reporting higher FoR. Furthermore, depressive mood severely impacts treatment adherence. Research indicates^[37]^ that FCR levels significantly influence patients’ health behaviors, hindering their ability to adopt proactive coping strategies for disease-related issues. This may create a vicious cycle: depression leads to negative coping strategies, which in turn generate FoR. Therefore, during clinical care, healthcare providers should conduct psychological assessments of postoperative patients and regularly monitor their mental state to alleviate their FoR.

#### 4.2.4. Perceived social support

The findings of this study indicate that perceived social support scores serve as a protective factor against FoR, suggesting that patients with higher perceived social support scores are less likely to experience FoR, similar to the findings of Zhen et al.^[11]^ Similarly, Koch-Gallenkamp et al^[38]^ found through a study of cancer patients that lower levels of social support serve as a predictor of FoR. Therefore, during clinical care, healthcare providers should strengthen health education and emotional support can alleviate patient concerns and reduce FoR levels.^[39]^

## 5. Limitations

This single-center, cross-sectional study may limit generalizability. Due to limitations in research conditions, this study did not classify CHD patients into subtypes such as unstable angina or myocardial infarction. It is recommended that future research might categorize CHD subtypes and conduct classified investigations. Large-scale, multicenter collaborative studies should be undertaken to enhance the representativeness of research findings. Additionally, trajectory analysis could be implemented to understand patients’ psychological status at different postoperative time points, thereby providing a basis for developing effective intervention strategies. This will provide a basis for the implementation of nursing interventions, thereby reducing the FoR level of patients and improving their quality of life.

## 6. Conclusion

Patients with CHD experience varying degrees of FoR after PCI. This study found that unemployed patients, those undergoing their first procedure, and those with depressive symptoms are more prone to developing FoR. Patients with strong social support are less likely to experience FoR. Based on the research findings, it is recommended that healthcare providers offer tailored support to patients according to their individual characteristics, thereby alleviating their psychological burden and reducing their FoR.

## Acknowledgments

The authors thank the cardiovascular units at the hospital for their support and help in recruiting patients. The authors are also grateful to all the respondents who participated in this study.

## Author contributions

**Methodology:** Qin Peng.

**Formal analysis:** Qin Peng.

**Visualization:** Qin Peng.

**Supervision:** Chaofeng Li.

**Investigation:** Yuxuan Shao, Xinyi Hou.

**Data curation:** Mengdie Hu.

**Writing – original draft:** Qin Peng.
